# Investigation of the Bioequivalence of Rosuvastatin 20 mg Tablets after a Single Oral Administration in Mediterranean Arabs Using a Validated LC-MS/MS Method

**DOI:** 10.3390/scipharm84030536

**Published:** 2016-06-30

**Authors:** Abdel Naser Zaid, Rowa Al Ramahi, Rita Cortesi, Ayman Mousa, Nidal Jaradat, Nadia Ghazal, Rana Bustami

**Affiliations:** 1 Department of Pharmacy, Faculty of Medicine & health Sciences, An-Najah National University, 44859 Nablus, P.O. Box 7, Palestine; rawa_ramahi@najah.edu (R.A.R.); nidaljaradat@najah.edu (N.J.); 2 Department of Life Sciences and Biotechnology, University of Ferrara, 44121 Ferrara, Italy; crt@unife.it; 3 R&D department Avalon Pharma (Middle East Pharmaceutical Industries Co. Ltd), 11372 Riyadh, Kingdom of Saudi Arabia; ayman@avalon.com.sa; 4 Naratech Pharma Consultancy, 11814 Amman, Jordan; nadia@naratech.net; 5 Pharmaceutical Research Unit, Complex No 19, Yajouz St 19, 11910 Amman, Jordan; rtbustami@pru.com.jo

**Keywords:** rosuvastatin calcium, bioequivalence, in vitro release, safety, efficacy

## Abstract

There is a wide inter-individual response to statin therapy including rosuvastatin calcium (RC), and it has been hypothesized that genetic differences may contribute to these variations. In fact, several studies have shown that pharmacokinetic (PK) parameters for RC are affected by race. The aim of this study is to demonstrate the interchangeability between two generic RC 20 mg film-coated tablets under fasting conditions among Mediterranean Arabs and to compare the pharmacokinetic results with Asian and Caucasian subjects from other studies. A single oral RC 20 mg dose, randomized, open-label, two-way crossover design study was conducted in 30 healthy Mediterranean Arab volunteers. Blood samples were collected prior to dosing and over a 72-h period. Concentrations in plasma were quantified using a validated liquid chromatography tandem mass spectrometry method. Twenty-six volunteers completed the study. Statistical comparison of the main PK parameters showed no significant difference between the generic and branded products. The point estimates (ratios of geometric mean %) were 107.73 (96.57–120.17), 103.61 (94.03–114.16), and 104.23 (94.84–114.54) for peak plasma concentration (C_max_), Area Under the Curve (AUC)_0→last_, and AUC_0→∞_, respectively. The 90% confidence intervals were within the pre-defined limits of 80%–125% as specified by the Food and Drug Administration and European Medicines Agency for bioequivalence studies. Both formulations were well-tolerated and no serious adverse events were reported. The PK results (AUC_0→last_ and C_max_) were close to those of the Caucasian subjects. This study showed that the test and reference products met the regulatory criteria for bioequivalence following a 20 mg oral dose of RC under fasting conditions. Both formulations also showed comparable safety results. The PK results of the test and reference in the study subjects fall within the acceptable interval of 80%–125% and they were very close to the results among Caucasians. These PK results may be useful in order to determine the suitable RC dose among Arab Mediterranean patients.

## 1. Introduction

Rosuvastatin calcium (RC) is a synthetic lipid-lowering drug. Its chemical structure is (3R,5S,6E)-7-[4-(4-fluorophenyl)-2-(*N*-methylmethanesulfonamido)-6-(propan-2-yl)pyrimidin-5-yl]-3,5-dihydroxyhept-6-enoic acid. The empirical formula for RC is (C_22_H_27_FN_3_O_6_S)_2_Ca and the molecular weight is 1001.14 g/mol. RC is a white amorphous powder that is sparingly soluble in water and methanol, and slightly soluble in ethanol. RC is a hydrophilic compound with a partition coefficient (octanol/water) of 0.13 at pH 7.0 [1,2].

It is a competitive inhibitor of coenzyme A reductase 3-hydroxy-3-methylglutaryl (HMG-CoA) which is a rate-limiting enzyme involved in converting HMG-CoA to mevalonate, a precursor of cholesterol. It is used in conjunction with a healthy diet and regular sport activities to treat patients with hyperlipidemia and mixed dyslipidemia, pediatric patients 10 to 17 years of age with heterozygous familial hypercholesterolemia, hypertriglyceridemia, primary dysbetalipoproteinemia (Type III hyperlipoproteinemia), homozygous familial hypercholesterolemia, slowing of the progression of atherosclerosis, and for primary prevention of cardiovascular diseases especially in men over 50 years old and women over 60 years old [1,2,3,4,5,6,7]. Oral administration of RC under fasting conditions results in peak plasma levels of the drug at 3 to 5 h while the elimination half-life is around 16–19 h [1,8].

Several studies have discussed the importance of pharmacogenetics in determining suitable drug doses of some medications and the related new guidelines to implement the pharmacogenetics in drug development and clinical practice [9,10]. In fact, recently several studies have been published on the effect of genetic variations on the safety and efficacy of drugs such as ticagrelor [11], adalimumab [12], and tamoxifen [13].

Statins were reported to be affected by genetic polymorphisms that may cause inter-individual variability in clinical safety and efficacy [14]. Rosuvastatin is one of these medications where some genetic determinants of LDL-C response were identified [15].

The populational PK studies revealed that plasma exposure to rosuvastatin was significantly higher in Asian subjects than in Caucasian subjects living in the same environment [1,16,17]. It is well-known that the highest available strength of rosuvastatin 40 mg has contraindications among Asian patients. In fact, a PK study conducted using 40 mg strength in order to compare rosuvastatin plasma concentrations among Caucasian and Asian subjects residing in the same area showed that the plasma concentrations in Chinese, Malay, and Asian-Indian subjects were nearly 2.3 fold, 2 fold, and 1.6 fold higher, respectively, than the Caucasian subjects [16]. In another study, plasma exposure to rosuvastatin was increased in six Asian groups compared with the Caucasian subjects. Peak plasma concentration (C_max_) and Area Under the Curve (AUC)_0→t_ were homogenous among five of the Asian groups (Chinese, Filipino, Korean, Vietnamese, and Japanese), and data were pooled. In the pooled Asian group, the AUC_0→t_ was approximately 1.7 fold greater than in Caucasian subjects. Exposure in Asian-Indian subjects was somewhat higher than that observed in Caucasian subjects, but was not homogeneous with the other Asian groups [17]. Due to complications with increased plasma concentrations of rosuvastatin, therapy should be initiated with 5 mg once daily in Asian populations [1,18].

RC tablets for oral administration are available in several strengths including 5, 10, 20, or 40 mg that are given once daily in order to meet patient requirements. Crestor^®^ tablets (AstraZeneca, London, UK) contain excipients such as microcrystalline cellulose, lactose monohydrate, tribasic calcium phosphate, crospovidone, magnesium stearate, hypromellose, triacetin, yellow ferric oxide, and red ferric oxide. Titanium dioxide USP is also included [1,2]. The same excipients were used in the formulation of the generic tablet product.

This study aimed to estimate the PK of rosuvastatin tablets of 20 mg strength and to assess the bioequivalence of the test and reference formulations Crestor^®^ (rosuvastatin) tablets 20 mg of AstraZeneca.

## 2. Materials and Methods

The study was a comparative, randomized, two-period, two-treatment, two-sequence, single dose, open-label, crossover bioequivalence study of generic RC 20 mg film-coated tablets versus the brand name drug Crestor^®^ 20 mg film-coated tablets given to healthy subjects under fasting conditions.

### 2.1. Volunteers and Clinical Protocol

The study was conducted by Arab Pharmaceutical Industry Consulting Co. Ltd. (Amman, Jordan) in accordance with the requirements of the declarations of Helsinki [19], the current Good Clinical Practice (GCP) Guidelines [20], and the International Conference Harmonization (ICH) Guidelines [21]. The study protocol and the informed consent forms were approved by the Institutional Review Board. Thirty adult male volunteers were recruited to participate in the study. Twenty-seven subjects completed the study and 26 subjects were evaluated for PK data (one subject was not able to reach the clinic for final evaluation due to severe weather conditions, so it was decided to exclude him from the final evaluation). The volunteers were healthy, Mediterranean Arabs aged between 18 and 50 years, body mass index was 18.5 to 30 kg/m^2^, and non-smokers or light smokers were enrolled in this study. The volunteers were subjected to a full medical and physical exam to confirm their healthy status and were not on any medication during the study period. A written informed consent form, which explained the nature of the study, was obtained from the volunteers. The volunteers were instructed to abstain from taking drugs one month before the study initiation, to stop consumption of caffeine and alcohol-containing beverages for at least 16 h prior to each drug administration and throughout the study period, and to fast for at least 10 h before drug administration.

The study used an open-label, randomized, two-period crossover design with an eight-day washout period between doses in the 30 healthy subjects under fasting conditions. The volunteers were randomly divided into two groups of 15 subjects each. The first group was given the reference brand and the second group was given the test formulation with a crossover after the washout period. On the morning of the study and prior to drug dosing, each volunteer gave a blood sample to serve as a blank for the drug assay. Then, each volunteer received an oral dose of the assigned tablet with 240 mL of water in the sitting position. During each period, blood samples were taken from each volunteer for the calculation of the PK parameters at pre- and up to 72 h post-drug dosing. Each sample volume was 8 mL; the samples were collected one hour before dosing and at the following time points: 0.50, 1.00, 1.50, 2.00, 2.50, 3.00, 3.50, 4.00, 4.50, 5.00, 5.50, 6.00, 7.00, 8.00, 10.00, 12.00, 16.00, 24.00, 36.00, 48.00, and 72.00 h after dosing. Blood samples were collected in tubes containing heparin, and centrifuged to separate the plasma fraction of the blood. The resulting plasma was immediately stored at −70 °C and analyzed by liquid chromatography tandem mass spectrometry (LC-MS/MS). Four hours after drug administration a standard lunch meal containing soup (no carrots), half a chicken, rice with mixed vegetables (no carrots), yogurt, a loaf of bread, salad (tomato and cucumber) was served and subjects had free access to water one hour after drug administration.

### 2.2. Chemicals and Reagents

RC working standard was supplied by Morepen Laboratories Ltd. (State Hemachal Pradesh, India) while the rosuvastatin-d6 internal standard (minimum purity is 98%) was supplied by TRC (North York, Canada). HPLC grade methanol and acetonitrile were purchased from Romil (Cambridge, UK), isopropanol was obtained from Carbon Group (Cork, Ireland), extra pure formic acid was obtained from Scharlau (Port Adelaide, Australia), diethylether was obtained from JHD (Guangzhou, China), and HPLC grade water was supplied by Sartorius Purified Water (Goettingen, Germany). Control human plasma was harvested from donors.

### 2.3. Tested Brand and Formulated Tablets

The generic film-coated tablets (20 mg/tablet) of batch no. 1309391 were obtained from Avalon Pharma, (Middle East Pharmaceutical Industries Co Ltd, Riyadh, Saudi Arabia). Crestor^®^ film-coated tablets (20 mg/tablet) of batch no. KD065 were obtained from AstraZeneca (Cheshire, UK).

### 2.4. Instruments and Chromatographic Separations

The Agilent 1200 liquid chromatography (LC) system (Agilent Technologies, Santa Clara, CA, USA) interfaced with an API 4000 tandem MS system (AB Sciex, Framingham, MA, USA) was used in the analysis. An isocratic system of the mobile phase consisted of (20:80:0.2) (V/V/V) (0.01 M ammonium acetate: methanol: acetic acid) and was used to elute the drug and the internal standard. The pH of the mobile phase was adjusted to 5.10 ± 0.20 and the stationary phase was (Agilent) Zorbax SB-C18 (2.1 × 50 mm), with a 1.8 μm column and an oven temperature of 40 °C. The injection volume was 30 μL with a flow rate equal to 0.200 mL/min. Detection settings were *m*/*z* (482.131/258.200) for RC and *m*/*z* (488.202/264.200) for the internal standard (rosuvastatin-d6). The retention times for the drug and internal standard were 0.95 min. A dissolution test apparatus (LabIndia, Mumbai, India) was used to assess the dissolution behavior and release of RC from the tablets. Electrospray ionization (ESI) interface in positive ion mode was chosen in order to improve the selectivity and the sensitivity required for this analysis.

### 2.5. Preparation of Standard and Working Solutions

A drug solution was prepared by dissolving 0.02041 g of RC (99.4%) in methanol. A final standard solution of 10.00 mL containing 7.807 μg/mL RC was prepared by diluting the stock solution using methanol. Internal standard working solution was prepared by diluting 0.030 mL from concentration 1 (C1) stock solution in a final volume of 25.00 mL methanol to make up a working solution containing 0.128 μg/mL. Prior to the extraction, 1.00 mL of each sample including: calibrators and quality control (QC) samples were spiked with 100 μL of internal standard (concentration 2 (C2): 0.128 μg/mL) to make up a final concentration of 11.636 ng/mL rosuvastatin-d6 (based on 1.100 mL final volume). Each sample was vortexed for 30 s. The internal standard was employed in order to ensure reliable quantitation of the sample.

### 2.6. Validation Procedures

The method was validated for its application to the analysis of RC in biological fluids by spiking it into blank plasma. The method was validated in accordance with the international guidelines of the Food and Drug Administration (FDA) [22] and European Medicines Agency (EMA) [23] for guidance on bioanalytical method validation and all the validation parameters including: linearity, accuracy, precision, and limit of quantitation (LOQ) which were calculated for the developed method.

The linearity assessment was performed using a series of nine standard plasma solutions previously spiked with rosuvastatin (calibrator) which were employed for constructing calibration curves covering a concentration range of 0.156–46.840 ng/mL. The accuracy and precision were determined by using a minimum of six replicates per concentration level. The LOQ was determined by injecting a series of diluted solutions with known concentrations.

In addition, stock solution stability in the mobile phase was assessed using two standard mixtures that are equivalent to the lower limit of quantification (LLOQ) and upper limit of quantification (ULOQ) concentrations with internal standard.

Short- and long-term matrix-based stability were assessed using two Quality Control Low (QCL) and Quality Control High (QCH) RC concentrations. Stability after freeze-thaw cycles was assessed using two sets of QC samples which were subjected to three freeze-thaw cycles (stability samples). The stability of the samples was compared to a freshly prepared calibration curve and was analyzed in a single run in comparison with a QC sample (comparison sample).

Whole blood stability was assessed by spiking whole blood samples with two different concentrations. The matrix effect was investigated for RC and the internal standard. The matrix factor (MF) was calculated in each lot of the matrix by calculating the ratio of the peak area in the presence of the matrix, to the peak area in the absence of the matrix (pure solution of the RC). The IS normalized MF was also calculated by dividing the MF of the analyte by the MF of the IS. The QC samples were used to evaluate the performance of the assay. They were prepared by spiking blank plasma with RC. The QC samples were prepared to have low, medium 1, medium 2, and high concentrations (RC: 6.72, 7.08, 11.11, and 14.88 ng/mL), respectively. Four QC samples were incorporated with each analysis run as unknown samples. The concentration in each QC sample was determined from the calibration curve and it was compared with the nominal concentration. The analysis run was accepted if at least three out of four QC samples were within 15% of the nominal concentration. No carryover or matrix effect was found.

### 2.7. In Vitro Release of RC from Tablets

High Performance Liquid Chromatography (HPLC) method was used in order to assess the amount of RC dissolved and released from generic and brand tablets. The employed analytical parameters are summarized in Table 1. The in vitro release of RC from the generic and brand tablets was conducted using the USP Apparatus (LabIndia, Mumbai, India) (II paddles) [24]. Twelve tablets were placed in the paddles of the dissolution apparatus (one tablet for each paddle). Each paddle contained 900 mL of dissolution media (0.05 M sodium citrate buffer pH 6.6 ± 0.05) and the speed of rotation in the paddles was 50 rpm. The temperature inside the paddles was held at 37 ± 0.5 °C during the entire period of the test. Samples (5 mL) were withdrawn at 10, 20, 30, and 45 min. Fresh blank dissolution medium (5 mL) was added to the paddles after each one was withdrawn. The samples were filtered through 0.45 μm filters. Precisely, politetrafluoroetileno (PTFE) membrane filters were used for acidic aqueous solutions, while nylon filters were used for the filtration of organic solutions. A sample of 0.25 μL was injected into the HPLC instrument.

Similarity and dissimilarity factors, *f*_2_ and *f*_1_, were calculated. The *f*_2_ factor indicates the closeness between two dissolution profiles and *f*_1_ indicates the difference between the two profiles according to equations (1) and (2) where *R_t_* and *T_t_* are the percentages of drug dissolved at each time point for the brand and generic products, respectively. An *f*_1_ value less than 15 means low dissimilarity, and an *f*_2_ value higher than 50 means significant similarity between the two products [25,26]. All required calculations were carried out using Microsoft Excel 2007 (Microsoft Corporation, Redmond, WA, United States.)

(1) $$f_{2}=50\cdot \log\left\{{\left[1+\frac{1}{n}\sum_{t=1}^{n}{\left(R_{t}-T_{t}\right)}^{2}\right]}^{-0.5}\times 100\right\}$$

(2) $$f_{1}=\left\{\left[\sum_{t=1}^{n}\left|R_{t}-T_{t}\right|\right]/\left[\sum_{t=1}^{n}R_{t}\right]\right\}\times 100$$

### 2.8. Pharmacokinetic and Statistical Analysis

The PK parameters were estimated using standard non-compartmental methods. C_max_ and the corresponding time of peak plasma concentration (t_max_) were taken directly from the data. The elimination rate constant (k_e_) was estimated from the slope of the semi-logarithmic plot of the terminal elimination phase of the plasma concentration-time curve. The equation t_1/2_ = ln2/k_e_ was used to calculate the elimination half–life time (t_1/2_). The areas under the drugs’ plasma concentration time curves from (AUC_0→last_) and the area to infinity (AUC_0→∞_) were calculated by using the linear trapezoidal method. Extrapolation to infinity was achieved by dividing the last measurable plasma concentration (C_last_) by the terminal rate constant k_e_. The AUC_0→inf_ was obtained from the sum of the estimated and extrapolated parts (AUC_0→inf_ = AUC_0→last_ + AUC_last→inf_). For the purpose of bioequivalence analysis, one-way analysis of variance (ANOVA) was used to assess the effect of formulations, periods, sequences, and subjects on AUC_0→last_, AUC_0→∞_, and C_max_. A commercially available software package version 5.1 (Thermo Scientific Kinetica, Waltham, MA, USA) was used for the calculation of the pharmacokinetic parameters, whereas SAS Analytics Pro 9.4 (SAS Institute Inc. Cary, NC, United States) was used in the statistical analysis of the pharmacokinetic data.

## 3. Results

### 3.1. Results of Validation Procedures

The developed and validated LC-MS/MS method was found to be rapid, sensitive, reproducible, and accurate for the analysis of RC in human plasma. The relationship between concentration and peak area ratio of RC/IS was found to be linear within the range 0.156–46.840 ng/mL for RC. The linear equation was y = 0.00780x − 0.003 with a correlation (R^2^) of 0.9975 during the course of the validation. The method was found to be sensitive with a LLOQ of 0.156 ng/mL.

Mean recovery was 90.58% for quality control low (QCL), 86.26% for quality control medium 1 (QCM1), 84.10% for quality control medium 2 (QCM2), and 83.13% for quality control high (QCH). IS recovery was 120.49%.

The method was accurate and precise to quantify the LLOQ of (0.156 ng/mL) with the precision of 17.65%. Short-term stability testing of RC in plasma proved that the drug is stable for up to 24 h at room temperature (RT). Stock solution short-term stability proved that the drug is stable up to 20 h at RT. A post-preparative stability study showed that the drug is stable for up to 40 h as summarized in Table 2.

### 3.2. Results of Dissolution Testing

The release of RC from the generic and brand tablet products in the recommended pH conditions was within the accepted levels; since *f2* and *f1* were 73 and 4, respectively.

### 3.3. Results of the Pharmacokinetic Study

**Figure 1:**
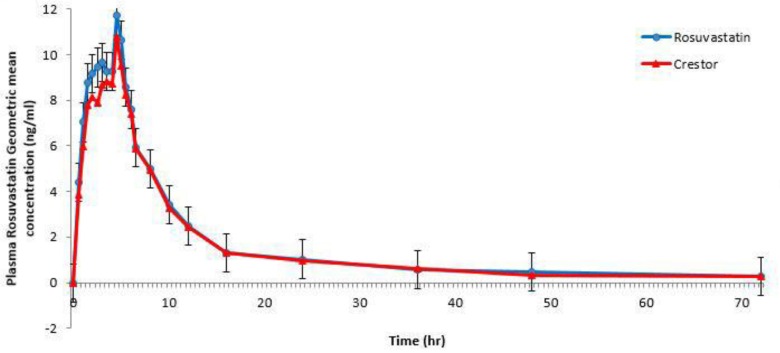
Plasma rosuvastatin geometric mean concentration (ng/mL) versus time (h) curve.

Both RC 20 mg film-coated tablets, the test tablets and Crestor^®^, were well-tolerated by all the subjects and they were discharged in good health. Figure 1 shows plasma concentrations of both brands indicating that the two brands are superimposable.

Table 3 shows a summary of the PK parameters for the two products of RC 20 mg film-coated tablets. The point estimates (ratios of geometric mean %) were 107.73 (96.57–120.17), 103.61 (94.03–114.16), and 104.23 (94.84–114.54) for C_max,_ AUC_0→last_, and AUC_0→∞_, respectively. No statistically significant difference between the two formulations was found. These PK parameter values lie within the FDA- and EMA-specified bioequivalence limit (80%–125%) [22,23]. Results in this part of the study showed equivalent clinical efficacy of the two brands. There were no serious or significant adverse events, with both formulations being well-tolerated when administered as a single dose and the obtained PK parameters were close to those conducted on Caucasian subjects (Table 4) [17].

## 4. Discussion

During the development phase of an oral solid dosage form, several pre-formulation and formulation trials and tests are carried out in order to achieve a generic product that can be interchangeable with the original brand in terms of efficacy and safety. Accordingly, in vitro dissolution in different pH media is conducted on the generic product and it must show a similar dissolution profile or overlapping to the reference brand. In many cases these tests cannot replace the in vivo tests which demonstrate the efficacy and safety of the generic product since it may contain different excipients. The in vitro dissolution study of the drug in three kinds of pH media was recommended by the ICH and other international guidelines in order to predict if the generic and brand tablet products could show comparable in vivo behavior. In fact, these tests are considered a very helpful indicator as pre-marketing surveillance for each batch of tablet products. However, for RC tablets, the dissolution conditions recommended by the FDA have pH 6.6 only, since the drug is acid-labile and degradation occurs under the other two dissolution conditions (pH 1.2 and 4.5, respectively). In this contest, the results of dissolution at the recommended pH 6.6 showed that the generic tablet product was comparable with the original brand, since *f2 and f1* were 73 and 4, respectively, and these values are within the recommended criteria of dissolution testing for RC generic tablets.

Statins are a class of drugs that have shown possible effects of genetic polymorphism on PK parameters [14]. There is a wide inter-individual response to statin therapy, and it has been hypothesized that genetic differences may contribute to these variations. However, genetic screening in order to guide the selection of lipid-lowering therapy might be a mirage since it would be a time- and money-consuming process [27]. An alternative tool in order to achieve personalized treatment could be the use of PK parameters. In fact, several studies have shown that PK parameters for statins are affected by race [16,17].

Previous studies have tested the bioequivalence of new generic RC tablets alone [8] or in combination with other drugs [28]. The aim of this study was to test the bioavailability of rosuvastatin 20 mg tablets produced by Avalon Pharma, versus the reference RC 20 mg (Crestor^®^) produced by AstraZeneca. The two dosage forms were administered to 30 fasting male volunteers in order to eliminate the influence of food on drug absorption.

The validated LC-MS method described above was utilized for quantification of RC in plasma samples. Analysis was successfully applied which provided the appropriate accuracy, sensitivity, linearity, precision, repeatability, and selectivity with high sample throughput and an economically-convenient procedure required for PK studies.

Regarding the efficacy of the generic product, statistical comparison of the main PK parameters, AUC_0→last_, AUC_0→∞_, C_max,_ and t_max_ clearly indicated no significant difference between the test and reference tablets, in any of the calculated PK parameters. The obtained values were compliant with the FDA and EMA requirements for the bioequivalence of generic drugs since the 90% CI for AUC_0→∞,_ AUC_0→last,_ and C_max_ mean ratios are within the 80%–125% interval [22,23].

Table 4 shows a summary of rosuvastatin pharmacokinetic parameters and the comparison in Asian, Caucasian (from reference [17]), and Arab subjects (current study) after administration of a single 20 mg dose. It is noticed that the AUC_0→last_ and C_max_ among subjects in the current study are comparable to those of Caucasian subjects, while the results of Asian subjects were higher. In the Birmingham et al. study [17], they tried to assess the influence of polymorphisms in SLCO1B1 (T521>C (Val174Ala) and A388>G (Asn130Asp) and in ABCG2 (C421>A (Gln141Lys) on exposure to rosuvastatin. Results showed that rosuvastatin exposure was higher in subjects carrying the SLCO1B1 521C allele compared with that in non-carriers of this allele. Similarly, exposure was higher in subjects carrying the ABCG2 421A allele compared with that in non-carriers [17]. Another study in Chinese subjects investigating the effect of the ABCG2 C421>A (Gln141Lys) polymorphism on rosuvastatin exposure has suggested that the 421A allele is significantly associated with higher Cmax and AUC than the 421C allele [29]. Genetic studies are recommended among Arabs and other races to explain the observed ethnic differences in exposure to rosuvastatin.

Safety is also important; in this study the administered drugs were tolerated and all the participating volunteers completed the study without showing any signs of adverse effects and were released in good health. The major limitation of the study as per all bioequivalence studies was the relatively small sample size and administration of a single dose in healthy male volunteers.

## 5. Conclusions

The statistical analysis of the results which were performed on AUC_0→last_, AUC_0→∞,_ and C_max_ using the ANOVA method showed that both test film-coated and reference tablets (Crestor^®^ 20 mg film-coated tablet) are bioequivalent. Both products have equivalent rates and extents of absorption exhibited by C_max_ and AUC_0→last_ ratios within the 80%–125% interval proposed by the FDA and the EMA. The PK results of the test and reference in the study subjects were very close to the results among Caucasians, accordingly, these PK results may be useful in order to determine the suitable rosuvastatin dose among Arab Mediterranean patients.

## Figures and Tables

**Table 1:** High Performance Liquid Chromatography (HPLC) analytical parameters for rosuvastatin calcium (RC).

**Mobile Phase**	*Buffer Solution Preparation*: Transfer 10 mL of Glacial Acetic Acid by Using a Volumetric Pipette in 5000 mL of Water. *Mobile Phase Preparation*: Mix 4500 mL of Buffer with 2500 mL of Methanol and 3000 mL of Acetonitrile.
**Diluent**	Dissolution media: 0.05 M of sodium citrate pH 6.6 (dissolve 74.9 g of sodium citrate and 45.9 g sodium chloride in 7000 mL of purified water, add 28 g sodium hydroxide and stir until dissolved and check pH). Adjust pH to 6.6 with sodium hydroxide or hydrochloric acid.
**HPLC**	Shimadzu system (Shimadzu corporation, Kyoto, Japan)
**Column**	Inertsil ODS-2 (25 cm × 4.6 mm) 5 μm.
**Detector**	UV detector @ 248 nm
**Flow Rate**	1.0 mL/min
**Injection Volume**	20 μL
**Column Oven Temperature**	25 °C
**Standard Solution Preparation**	Standard solution contains: 0.0222 mg/mL of rosuvastatin.
**Sample Solution Preparation**	After the end of the run or at a certain sampling time point, withdraw 10 mL from each vessel and filter through a 0.45 μm filter.
**Run Time**	15 min
**Filter**	a. 0.45 μm PTFE (saturate the filter, discard the first 3 mL from the standard solution). b. 0.45 μm nylon (saturate the filter, discard the first 3 mL from the standard solution).

**Table 2:** Summary results of short- and long-term stability of RC in plasma and chromatographic solutions.

**Short-Term Stability (Plasma)**		Up to 24 h at RT.
**Short-Term Stability (Stock Solution)**		Up to 20 h at RT (for RC and rosuvastatin-d6).
**FTC Stability**		Up to 4 cycles at −70 °C. Up to 4 cycles at −20 °C.
**Long-Term Stability (Stock Solution)**		Up to 30 days for RC and rosuvastatin-d6 at −20°C.
**Long-Term Stability (Plasma)**		Up to 60 days at −70 °C. Up to 60 days at −20 °C.
**Post-Preparative**	***Injection Phase***	Up to 44 h at 5 °C. Up to 44 h at −20 °C.
	***Dry Phase***	
**Whole Blood Stability**		% of change between QCL and QCH = −0.48
**Matrix-Dilution Integrity**		Samples above ULOQ (46.840 ng/mL) and up to 140.520 ng/mL can be diluted with a dilution factor of 3.
**Matrix Effect**		CV% of rosuvastatin-d6 normalized was 8.75% and 1.93%.

RT: room temperature; QCL: quality control low; QCH: quality control high; ULOQ: upper limit of quantification: CV: coefficient of variation.

**Table 3:** Summary of calculated pharmacokinetic parameters.

Efficacy Results Summary	Parameters (unit)	Test Rosuvastatin		Reference Crestor	
As Geometric Means (Ranges) for C_max_ and AUC Ratios	C_max_ (ng/mL)	12.059		11.194	
		5.973	37.323	3.495	35.545
	AUC_0→last_ (ng·h/mL)	104.263		100.635	
		40.335	254.681	30.152	263.448
	AUC_0→∞_ (ng·h/mL)	110.786		106.294	
		42.642	267.086	33.354	271.362
As Medians (Ranges) for t_max_ and t_1/2_	t_max_ (h)	4.50		4.50	
		0.50	5.50	0.50	5.0
	t_1/2_ (h)	6.80		7.73	
		4.87	21.07	3.34	22.26
Bioequivalence Results Summary	Parameter	Point estimate (ratio of geometric mean %)	Lower limit %	Upper limit %	CV%
	C_max_	107.73	96.57	120.17	23.34
	AUC_0→last_	103.61	94.03	114.16	20.66
	AUC_0→∞_	104.23	94.84	114.54	20.09

C_max_: peak plasma concentration; AUC: Area Under the Curve; t_max_: time of peak plasma concentration; t_1/2_: elimination half–life time

**Table 4:** Summary of RC pharmacokinetic parameters and comparison in Asian, Caucasian, and Arab subjects after administration of a single 20 mg dose.

	A	B	C	D	E	F	G	H	J	K
AUC_0→t_ (ng·h/mL) Gmean Ratio *	202 1.74	207 1.79	213 1.84	146 1.26	191 1.64	205 1.76	193 1.66	116 1.00	104 0.90	101 0.87
Cmax (ng/mL) Gmean Ratio*	22.0 1.85	22.4 1.89	23.3 1.97	15.3 1.29	20.5 1.72	20.2 1.7	23.6 1.98	11.9 1.00	12.1 1.02	11.2 0.94
t_max_ (h) Median	4.00	4.00	3.00	5.00	3.54	5.00	5.00	4.50	4.50	4.50

A: Pooled Asian, B: Chinese; C: Filipino; D: Asian-Indian; E: Korean; F: Vietnamese; G: Japanese, H: Caucasian; J: Arab Test; K: Arab Reference; Results of pooled Asian, Chinese, Filipino, Asian-Indian, Korean, Vietnamese, Japanese, and Caucasian are from reference 17. * Ratio of Asian subgroups and Arabs to Caucasian subjects derived from the geometric mean.
